# Delaying first birth: an analysis of household survey data from rural Southern Tanzania

**DOI:** 10.1186/s12889-017-4069-2

**Published:** 2017-01-31

**Authors:** Yovitha Sedekia, Rose Nathan, Kathryn Church, Silas Temu, Claudia Hanson, Joanna Schellenberg, Tanya Marchant

**Affiliations:** 1 Ifakara Health Institute, P.O BOX 78373, Dar-es-Salaam, Tanzania; 2 Department of Disease Control, London School of Hygiene and Tropical Medicine, Keppel Street, London, WC1E 7HT UK; 3 Department of Population Health, London School of Hygiene and Tropical Medicine, Keppel Street, London, WC1E 7HT UK; 4 Department of Public Health Science (Global Health), Karolinska Institutet, Stockholm, Sweden

**Keywords:** Adolescents, Contraceptives, Delayers of first birth, Family planning use, Maternal and child health, Unmet need, Tanzania

## Abstract

**Background:**

Currently, family planning metrics derived from nationally-representative household surveys such as the Demographic and Health Surveys (DHS) categorise women into those desiring to space or limit (permanently stop) births, or according to their age in the case of young women. This conceptualisation potentially ignores a large and growing group of young women who desire to delay a first birth. This study uses household survey data to investigate the characteristics and needs for family planning of women who want to delay their first birth.

**Methods:**

The research was conducted in two rural districts in southern Tanzania (Tandahimba and Newala), and nested within the Expanded Quality Management Using Information Power (EQUIP) study. Data were collected as part of a repeated cross sectional household survey conducted between September 2013 and April 2014. The socio-demographic characteristics, including parity, contraceptive practices and fertility intentions of 2128 women aged 13–49 were analysed. The association between women’s life stages of reproduction (delayers of first birth, spacers of subsequent pregnancies and limiters of future birth) and selected contraceptive outcomes (current use, unmet need and demand for modern contraceptives) was assessed using the point estimates and 95% confidence intervals for each indicator, adjusted for the survey design.

**Results:**

Overall, four percent of women surveyed were categorised as ‘delayers of first birth’, i.e. sexually active but not started childbearing. Among this group, the majority were younger than 20 years old (82%) and unmarried (88%). Fifty-nine percent were currently using a modern method of contraception and injectables dominated their contraceptive use. Unmet need for contraception was higher among delayers (41%; 95% CI 32–51) and limiters (41%; 95% CI 35–47) compared to spacers (19%; 95% CI 17–22).

**Conclusions:**

Delayers of first birth have very high unmet needs for modern contraceptives and they should be routinely and separately categorised and measured within nationally-representative surveys such as Demographic and Health Survey and Multiple Indicator Cluster surveys. Acknowledging their unique needs could help catalyse a programmatic response.

## Background

Evidence that family planning affects the life course of women from the moment of their own birth through to menopause is abundant and compelling [1–6]. Since family planning improves perinatal and child survival outcomes through lengthening the inter-pregnancy interval [3], children born to parents who had the power and means to decide on the number and spacing of pregnancies tend to be healthier, do better in school and get opportunities to earn higher incomes [4]. Furthermore, use of contraceptives can help both adolescent and post-adolescent women to start child-bearing later, thus allowing them to complete their education and offering opportunities to engage in income-producing activities [4, 7].

Currently, family planning metrics derived from nationally-representative household surveys such as the Demographic and Health Surveys categorise women into those desiring to space or limit (permanently stop) birth [8–11], or according to their age (particularly for young women aged 15–24 years). Those wishing to delay their first birth are not readily identifiable as a group with a distinct profile and their specific reproductive needs may be neglected even though this group of women are likely to become increasingly important. For example, an analysis of DHS data revealed unmet need for family planning to be highest amongst young married women with no children than those with a child [12]. In sub-Saharan Africa this finding is consistent with evidence that age at first sex is falling [13], age at first marriage is increasing [14] and women are more empowered to demand education and rights to determine the timing of a pregnancy [15, 16].

In Tanzania, public policies and strategies are in place for the achievement of universal access to family planning, backed-up by strong political commitment [17–20]. Men and women in the country including young people (10–24 years of age) regardless of parity, marital status, creed, race, or sexual preference are legally eligible to access accurate and complete family planning information, education and services without the need for parental or spousal consent [21]. Tanzania is also a Family Planning 2020 focus country, a global initiative that aims to expand contraceptive use to 120 million additional women and girls by 2020 [22, 23]. Girl’s enrolment into secondary school has increased over the last decade as a consequence of government commitment to provide free primary and secondary education [24, 25].

In the context of increased global attention to family planning and reproductive rights, and to the education of girls, it is important to understand the needs of girls and women who are sexually active but who wish to delay their first birth. Using data from the high fertility setting of Tanzania, we estimated how many sexually active married or unmarried women aged 13–49 years expressed a preference to delay their first birth, described their characteristics and examined the family planning outcomes for this group of ‘delayers’, contrasting them to spacers of subsequent pregnancies and limiters of future birth.

## Method

### Study setting

Detailed information about the study setting is provided elsewhere [26, 27]. Briefly, this research was carried out in two rural districts of Mtwara region in southern Tanzania: Tandahimba and Newala. The research was nested within the Expanded Quality Management Using Information Power (EQUIP) study [26, 27].

Tandahimba and Newala districts in Mtwara region-Southern Tanzania, where this study was carried out, cover an estimated population of over 400,000 people served by 63 health facilities [26–28], and is characterised as predominantly rural, having limited infrastructure [29] and high maternal and newborn mortality rates of 712 per 100,000 live births [30] and 31 deaths per 1000 live births respectively [31]. Makonde is the dominant ethnic group in the study area and over 90% of the population depends on agricultural activities which include cash (cashew nuts, sesame and groundnuts) and food crops (cassava, maize, rice and sorghum) [29]. The most recent Demographic and Health Survey (2010) estimated the Mtwara region to have a total fertility rate of 4.4, median age at first birth of 19 years, and high estimates for use of modern family planning methods (37%) in comparison to the rest of Tanzania mainland (27%) in 2010 [31], measured among married women aged 15–49 years. Among current users of any family planning method, 25% were using for spacing and 13% for limiting; and among those with unmet need (24%), half was for spacing and half for limiting (at 12% respectively) [31].

### Study design and participants

Data were collected as part of the repeated cross sectional household surveys conducted by the EQUIP study between September 2013 and April 2014. Full details about the survey methods are reported elsewhere [26]. In short, each month, in each district, a representative sample of 10 household clusters (defined as sub-villages) each of 30 households was drawn. For each district, sub-villages were listed and the number of households in each sub-village cumulated then 10 clusters selected with probability proportional to the total number of households in the district. The survey applied modular survey tools compatible with DHS and Multiple Indicators Cluster surveys to estimate indicators across the reproductive, maternal and newborn health continuum among resident women aged 13–49 years. All household heads and resident women (aged 13 to 49 years) who gave consent were interviewed. Household heads were interviewed about residents and household characteristics; whereas, women aged 13–49 years were interviewed about maternal and newborn health care and family planning knowledge and services.

### Data processing and analysis

Data were analysed using STATA 13 [32]. For the purpose of this analysis, sexually active women included married (including cohabiting) and unmarried women aged 13–49 years who reported having had sexual intercourse in the past three months. Women’s life stages of reproduction were categorised as follows: (1) delayers of first birth (nulliparous who on survey day reported a preference to delay their first birth for at least two years or more), (2) spacers of subsequent pregnancies (parous who on survey day desired to wait for at least two years or more before having another child), (3) limiters of future birth (parous who had reached their desired family size and on survey day reported that they did not desire any subsequent children), (4) desire child soon (nulliparous and parous who on survey day desired to have a child within two years), and (5) infecund (women who on survey day reported that cannot get pregnant or had never been pregnant in the past five years and had never used contraceptives) as indicated in Table 1.

**Table 1:** Women’s life stages of reproduction

Women’s life stages of reproduction	Sexually active (married and unmarried) women aged 13–49 who at the time of the interview:	N	% (95% CI)
	(*N* = 2128)		
Delayers of first birth (“delayers”)	• Were nulliparous (including women who reported previous pregnancy but no live birth), not currently pregnant, and their preference was to delay their first birth for at least two years or more	83	4 (3–5)
Spacers of subsequent pregnancies (“spacers”)	• Had started child bearing (including current pregnant women) and desired to wait at least for two years or more before having another child	790	37 (35–40)
Limiters of future birth (“limiters”)	• Had reached their desired family size (including current pregnant women) and did not desire any subsequent children	409	19 (17–21)
Desire child soon	• Have started childbearing, had at least one child and at the time of interview wanted a child within two years	675	32 (30–34)
	• Have never had a child and at the time of interview they were not pregnant and wanted child within two years	108	5 (4–6)
Infecund	• Self-reported that cannot get pregnant or married for the past five years and never been pregnant, never used contraceptives, currently not pregnant and currently not using contraceptives	63	3 (2–4)

Modern contraceptives were defined according to Hubacher, [33] and included short acting contraceptives (pills, injectables and condoms), long acting reversible contraceptives (implants and intrauterine devices and systems (IUDs) and permanent contraceptive methods (male and female sterilization). We applied the definition of unmet need for contraceptives as per Bradley S et al. [34], but restricted to modern contraceptive methods as per Westoff [10]. Delayers of first birth who on survey day reported a preference to delay their first birth for at least two years or more but were currently not using modern contraceptives were classified as having unmet need.

Percentages and 95% confidence intervals were used to show distribution of women in various background characteristics including quintiles of socio-economic status that was derived from a wealth index constructed using principal components analysis of asset ownership. The association between women’s life stages of reproduction (delayers of first birth, spacers of subsequent pregnancies and limiters of future birth) and selected contraceptive outcomes (current use, unmet need and demand for modern contraceptives) was assessed using the point estimates and 95% confidence intervals for each indicator, adjusted for the survey design using “*svy*” commands in STATA.

## Results

### Study population

Between September 2013 and April 2014 a total of 4723 households were sampled across both districts, 3820 resident women aged 13–49 years identified, of whom 3578 were interviewed. Among the 3578 respondents, 2128 (59%) were sexually active in the last three months and included in this analysis. Of these, 1772 (83%) were currently married or cohabiting, mean parity was 3 births (range 0–11), 13% had no education, 97% were Muslim, and 92% were of the Makonde ethnic group (Table 2).

**Table 2:** Characteristics of study sample

Background characteristics	All women 13–49 years (*N* = 3578)		Sexually active (married and unmarried) women 13–49 years included in the analysis (*N* = 2128)	
	n	% (95% CI)	n	% (95% CI)
Age groups (yrs)				
13–14	205	6 (5–7)	10	<1 (0–1)
15–19	529	15 (14–16)	182	9 (7–10)
20–24	491	14 (12–15)	297	14 (12–16)
25–29	466	13 (12–14)	312	15 (13–17)
30–34	527	15 (13–16)	355	17 (15–18)
35–39	519	15 (13–16)	344	16 (15–18)
40–44	504	14 (13–15)	361	17 (16–19)
45–49	337	9 (8–11)	267	13 (11–14)
Median age				
Median	3578	30 (IQR 21–39)	2128	33 (IQR 25–41)
Marital status				
Currently married	2344	66 (64–67)	1713	80 (79–82)
Cohabiting	82	2 (2–3)	59	3 (2–4)
Divorced/separated	439	12 (11–13)	212	10 (9–11)
Widow	22	1 (0–1)	4	<1 (0–0)
Single	691	19 (18–21)	140	7 (6–8)
Education				
No education	463	13 (11–15)	286	13 (12–16)
Some primary	474	13 (12–15)	243	11 (10–13)
Completed primary	604	73 (70–75)	1574	74 (72–77)
Some secondary or higher	30	1 (0–2)	20	1 (0–2)
Religion				
Muslim	3459	97 (95–98)	2054	97 (95–98)
Others	119	3 (2–5)	74	3 (2–5)
Ethnicity				
Makonde	3338	93 (91–95)	1965	92 (90–94)
Others	238	7 (5–9)	162	8 (6–10)
Household socio-economic status				
Q1 (most poor)	420	12 (10–13)	306	10 (8–11)
Q2	586	16 (15–18)	334	16 (14–18)
Q3	692	19 (18–21)	414	19 (18–21)
Q4	914	26 (23–28)	5611	26 (24–29)
Q5 (least poor)	966	27 (24–30)	613	29 (26–32)
Parity				
Mean parity	3578	3 births (range 0–11)	2128	3 births (range 0–11)
Total	3578	100	2128	59

*IQR Inter*-*quartile range*, *25th and 75th percentiles*

### Women’s life stages of reproduction

The distribution of women across the life stages of reproduction is shown in Table 1. Four percent (95% CI 3–5) had never had a child and reported a preference to delay their first birth for at least two years or more (“delayers”), 37% (95% CI 35–40) had started child bearing and desired to wait for at least two years or more before having another child (“spacers”), 19% (95% CI 17–21) did not desire any subsequent children (“limiters”), 32% (95% CI 30–34) had at least one child and wanted another child in the next two years, 5% (95% CI 4–6) had never had a child and wanted a child in the next two years and 3% (95% CI 2–4) were infecund.

### Characteristics of the delayers, spacers and limiters

Table 3 shows selected background characteristics of delayers of first birth and contrasts these to those of spacers and limiters. As expected, women categorised as delayers were younger on average than spacers and limiters, although of interest was that 18% of them were 20 years or older and 12% (95% CI 7–20) of the delayers were currently married or cohabiting. We found no difference in distribution by level of education attained, or other key socio-demographic characteristics (religion, ethnicity or socio-economic status of households) between women’s life stages of reproduction (delayers, spacers and limiters).

**Table 3:** Characteristics of the delayers, spacers and limiters aged 13–49 years

	Sexually active ^a^ (married and unmarried) women 13–49 years who are: (*N* = 2128)					
Background Characteristics	Delayers (*N* = 83)		Spacers (*N* = 790)		Limiters (*N* = 409)	
	n	% (95% CI)	n	% (95% CI)	n	% (95% CI)
Age group (yrs)						
13–14	9	11 (6–18)	0	0	0	0
15–19	59	71 (61–79)	60	8 (6–10)	0	0
20–24	10	12 (6–23)	168	21 (18–25)	11	3 (2–5)
25–29	1	1 (0–8)	157	20 (17–24)	25	6 (4–9)
30–34	3	4 (1–11)	170	22 (18–25)	41	10 (7–13)
35–39	1	1 (0–8)	121	15 (13–18)	57	14 (11–18)
40–44	0	0	73	9 (7–12)	126	31 (27–35)
45–49	0	0	39	5 (4–7)	148	36 (31–42)
Median age						
Median	83	16 (IQR 15–18)	790	30 (IQR 24–36)	409	42 (IQR 37–46)
Marital status						
Currently married	9	11 (6–19)	647	82 (79–85)	354	87 (82–90)
Cohabiting	1	1 (0–8)	23	3 (2–4)	15	4 (2–7)
Divorced/separated	3	4 (1–10)	90	11 (9–14)	40	10 (7–13)
Widow	0	0	0	0	0	0
Single	70	84 (77–90)	30	4 (3–5)	0	0
Education						
No education	6	7 (3–15)	99	13 (10–15)	79	19 (15–24)
Some primary	5	6 (3–14)	90	11 (9–14)	54	14 (10–17)
Completed primary	72	87 (77–93)	590	75 (71–78)	272	67 (62–71)
Some secondary or higher	0	0	10	1 (0–3)	2	<1 (0–2)
Religion						
Muslim	80	96 (89–99)	768	97 (96–98)	388	95 (92–97)
Others	3	4 (1–11)	22	3 (2–4)	21	5 (3–8)
Ethnicity						
Makonde	78	94 (84–98)	730	92 (89–95)	376	92 (87–95)
Others	5	6 (2–16)	60	8 (5–11)	33	8 (5–16)
Household socio-economic status						
Q1 (most poor)	10	12 (7–20)	85	11 (9–13)	37	9 (6–13)
Q2	18	22 (14–31)	126	16 (13–19)	58	14 (11–18)
Q3	18	22 (14–31)	144	18 (16–21)	97	24 (19–29)
Q4	20	24 (16–35)	220	28 (24–32)	107	26 (22–31)
Q5 (least poor)	17	20 (13–30)	215	27 (24–31)	110	27 (22–32)
Parity						
Mean parity	83	n/a	790	3 births (range 1–8)	409	4 births (range 1–11)
Total	83	4 (3–5)	790	41 (39–43)	409	19 (18–21)

*n*/*a not applicable*

*IQR Inter*-*quartile range*, *25th and 75th percentiles*

^a^ *Characteristics of sexually active women desiring a child*/*another child soon within 2 years and infecund women are excluded from the table*

### Current use, unmet need and demand for modern contraceptives among delayers, spacers and limiters

Table 4 presents current use, unmet need and demand for modern contraceptives by women’s life stages of reproduction (delayers, spacers, limiters). Fifty nine percent (95% CI 49–68) of delayers were currently using a modern method of contraception, similar to the proportion among spacers (65%; 95% CI 62–68) and limiters (53%; 95% CI 47–59). However, the proportion of unmet need for modern contraceptives was higher among delayers (41%; 95% CI 32–51) and limiters (41%; 95% CI 35–47) than spacers (19%; 95% CI 17–22). Total demand for modern contraceptives was high for all groups being universal amongst delayers (as indicated by their definition of not wanting a birth), 94% amongst limiters (95% CI 91–96) and 84% among spacers (95% CI 81–86).

**Table 4:** Percentage of current use, unmet need and demand for modern contraceptive methods among delayers, spacers and limiters aged 13–49 years

Contraceptive Outcome	Delayers (*N* = 83)		Spacers (*N* = 790)		Limiters (*N* = 409)	
	n	% (95% CI)	n	% (95% CI)	n	% (95% CI)
Current use of modern contraceptive	49	59 (49–68)	512	65 (62–68)	218	53 (47–59)
Unmet need for modern contraceptives	34	41 (32–51)	152	19 (17–22)	167	41 (35–47)
Demand for modern contraceptives	83	100	664	84 (81–86)	385	94 (91–96)

### Types of modern contraceptives used

**Fig. 1:**
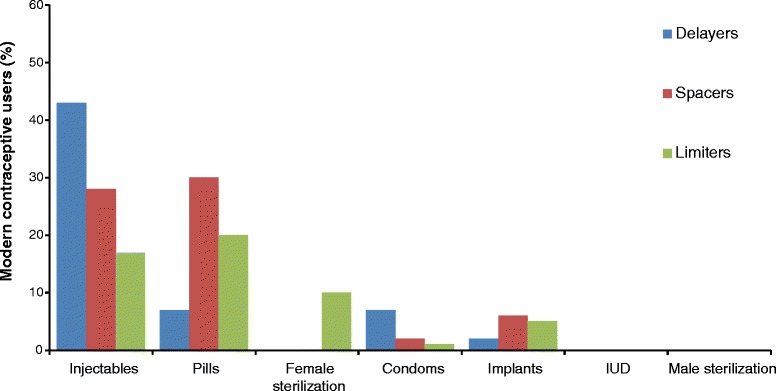
Percentage of modern contraceptive users by type of method used among delayers, spacers and limiters aged 13–49 years

Figure 1 presents the different types of modern contraceptives currently used by women according to their reproductive stage. Injectables (26%; 95% CI 23–29) and pills (25%; 95% CI 22–28) were the most commonly used methods, followed by implants (5%; 95% CI 4–7), female sterilization (3%; 95% CI 2–4) and condoms (2%; 95% CI 1–3). Use of injectables was higher among delayers (43%; 95% CI 34–53) than spacers (28%; 95% CI 24–31) or limiters, (17%; 95% CI 15–24). Condoms were also more commonly used by delayers (7%; 95% CI 3–15) than limiters (1%; 95% CI 1–3) or spacers (2%; 95% CI 1–3).

## Discussion

In this study we highlighted a small but important group of sexually active women aged 13–49 years who had not started childbearing and wanted to delay their first birth. The majority of this group are younger than 20 years old and unmarried. More than half were currently using a modern method of contraception, and injectables dominated their contraceptive use. Despite the fact that in our findings, only four percent of women were delayers of first birth, this is equivalent to approximately 281,778 women aged 13–49 years in the whole country of Tanzania in 2010 [35]. Taking our findings on 41% of delayers having unmet need for family planning, this equates to 115,529 women in the whole country of Tanzania who want to delay their first birth but have an unmet need for modern contraceptives.

Delayed first birth, delayed marriage or delayed sexual debut all have the potential to lead to lower fertility [36]. In high and middle income countries, where secondary education is universal, women delay their first birth well beyond the adolescent years [37–39]. For example, in the United Kingdom the average age of women at first birth in 2013 was 30 years [40], and data suggests that first births to women aged 35–39 years and 40–44 years continue to rise [41]. While important cultural differences should persist, similar trends in delayed childbearing are likely to occur in sub-Saharan Africa as school enrolment and income-earning opportunities for women increase, and the continent moves away from widespread high fertility norms that places high expectations on young women to start childbearing, maintain family lineage and provide labour [24, 42].

Injectables are the most commonly used contraceptive method in East Africa (including Tanzania) accounting for over 40% of contraceptive use [43, 44], and were the most commonly used method by delayers of first birth in our study. Of concern is that contraceptive discontinuation rate for users of injectables and pills has been reported to be high, leading to part of the explanation for increases in unmet need in women who have tried either injectables or pills but discontinued their use without switching to another [45]. For young people who may engage in intercourse infrequently, there is clearly a need to provide alternatives, including long-acting reversible contraceptives such as implants or IUDs which can offer long-term contraceptive protection. But currently in Tanzania long-acting reversible contraceptives are not widely available throughout the country [46].

One of the strengths of our study was that it included women aged 13–14 years who are typically not included in the sampling frame of surveys such as DHS. Our analysis indicated that 11% of the sexually active delayers were aged 13–14 years and available data suggests that age at first sex is decreasing [13] and unintended pregnancies continue to exist among young teenage women in Tanzania [47, 48]. This is not a problem for Tanzania alone. The State of World Population report 2013 stated that of 7.3 million (19%) births to women under 18 years in developing countries, two million (3%) were to girls who were 14 years or younger [49] and who are most at risk of grave long-term health and social consequences from pregnancy. They are also likely to be excluded in the family planning policies and other reproductive health services [7].

Our study had three important limitations. First, with regard to definition of family planning indicators used by DHS, in addition to including women aged 13–49 not 15–49 years, we also included a recall period of three months not four weeks for sexual activity because of concerns that young unmarried people may have less frequent sexual relations than other women but still be at risk because of a lack of protection, and ignored because of a lack of attention. However this may have had the effect of over-estimating the number of women classified as recently sexually active, although our findings on family planning use for spacing and limiting were consistent with existing estimates. Further, we did not have data with which to directly categorise women as menopausal or not, although this was unlikely to affect our findings on delaying first birth.

Second, despite intensive training and supervision of enumerators the risk of social desirability bias cannot be eliminated, particularly with regards to reporting sexual activities among unmarried and young women, which may have led to an underestimation of the number of women at risk. A study from Ethiopia suggested that unmarried women aged 13–24 years might only report half of sexual activities but over exaggerated on contraceptive and condom use [50]. Third, recall bias on timing for last sexual activity may also have been present, especially among unmarried women; and errors in age reporting cannot be discounted.

## Conclusions

In conclusion, our study demonstrates that even in this rural environment a small but important proportion of sexually active women would like to delay their first birth. Nearly all these women had some formal education, and all had a demand for modern contraceptives, but nearly half had an unmet need for contraception suggesting they are not currently well served by family planning programmes. We propose that delayers of first birth should be consistently categorised, using nationally representative survey data, preferably from a younger age than currently assessed, and their needs addressed in policy and programme formulation.

## Abbreviations

CI: Confidence interval
DHS: Demographic health surveys
FP: Family planning
